# Reverse Shoulder Arthroplasty in Patients with Rheumatoid Arthritis: A Nationwide Analysis

**DOI:** 10.3390/jcm14030994

**Published:** 2025-02-04

**Authors:** Andrew G. Beauperthuy, Nadia F. Linton, Peter A. Falgiano, Kevin L. Mekkawy, Hugo C. Rodriguez, Ashim Gupta

**Affiliations:** 1 Department of Orthopaedic Surgery, Larkin Community Hospital, South Miami, FL 33143, USA; ab3941@mynsu.nova.edu (A.G.B.); peter.falgiano@gmail.com (P.A.F.); kevin.mekkawy@gmail.com (K.L.M.); drhugocrodriguez@gmail.com (H.C.R.); 2 Miami Orthopaedic Research Foundation, Miami, FL 33186, USA; 3 Touro College of Osteopathic Medicine, New York City, NY 10027, USA; nlinton@student.touro.edu; 4 Hospital for Special Surgery, West Palm Beach, FL 33401, USA; 5 Future Biologics, Lawrenceville, GA 30043, USA

**Keywords:** rheumatoid arthritis, reverse shoulder arthroplasty, arthroplasty, outcomes, complications

## Abstract

**Introduction:** Reverse shoulder arthroplasty (RSA) was originally developed for treating rotator cuff arthropathy but is now commonly used for rheumatoid arthritis (RA)-related shoulder degeneration. While previous studies have identified RA to be a risk factor for complications following total shoulder arthroplasty, its specific impact on RSA outcomes remains unclear. This study aims to evaluate the post-operative medical and implant-related complications, and healthcare utilization, among RA patients undergoing RSA. **Methods:** A retrospective analysis of patients undergoing RSA was conducted using a national administrative claims database from 2010 to 2023. Patients who underwent RSA with and without RA were identified using corresponding diagnoses and procedural codes. Patients with RA who underwent RSA had propensity score matched to a control on a 1:5 basis. The control group consisted of patients who did not have RA and underwent RSA for any other indication. **Results:** A total of 7232 of RSA patients with RA were matched to 36,054 control patients. The RA cohort had significantly higher rates of 90-day medical complications when compared to the control (*p* < 0.001), with the highest rates in urinary tract infections (OR: 9.69), pulmonary embolisms (OR: 9.69), and the need for blood transfusions (OR:9.41). Patients with RA had significantly greater odds of developing all implant-related complications within 2 years compared to the control group (*p* < 0.001). This cohort also had significantly higher fall rates (*p* < 0.001) and mean lengths of stay (3.42 vs. 2.0 days, *p* < 0.0001). **Conclusions:** RSA patients with prior diagnoses of RA face a higher risk of implant-related and medical complications, falls, and prolonged hospital stays compared to the control. These findings suggest that RA is an independent risk factor for reverse total shoulder arthroplasty. Therefore, these patients should be closely monitored post-operatively to reduce complications, cost of care, and length of stay. Level of Evidence: III, retrospective case–control study

## 1. Introduction

Rheumatoid arthritis (RA) is a chronic autoimmune condition characterized by inflammatory arthritis and extra-articular involvement. In advanced disease, synovial joints undergo destruction by bony erosion and loss of articular cartilage. Extra-articular disease affects the lungs, eyes, heart, kidneys, skin, and digestive tract, and is associated with increased mortality [1]. As of 2020, an estimated 17.6 million people worldwide had RA, and this value is expected to double by 2050 [2].

Reverse shoulder arthroplasty (RSA) is a semi-constrained prosthesis in which the ball and socket configuration of the normal shoulder is reversed: a concave proximal humerus articulates with a glenosphere on the proximal humerus. Since being initially developed for treating rotator cuff arthropathy, the list of indications for this prosthesis has grown substantially. RSA is now commonly used to treat primary glenohumeral osteoarthritis, inflammatory arthritis, and proximal humerus fractures. Excellent clinical outcomes, an aging population, and improved implant design have also contributed to the increase in the number of RSAs performed annually [3,4]. From 2011 to 2020, there was a 394% surge in cases of total shoulder arthroplasty performed, of which RSA increased from 31% to 75% [5].

The prior literature has identified RA as an independent risk factor for complications following both total joint [6] and total shoulder arthroplasty [7]. However, the impact of RA on outcomes specifically pertaining to RSA has not been described extensively. An improved understanding of outcomes and healthcare utilization for patients with RA undergoing RSA will allow surgeons to make more informed decisions regarding patient care and help to manage expectations post-operatively. Considering the upward trend in the utilization of RSA, surgeons are increasingly likely to encounter RA patients who are indicated for this operation.

The objective of this study was to assess (1) post-operative medical complications, (2) implant-related complications, and (3) healthcare utilization in patients with RA undergoing RSA.

## 2. Materials and Methods

### 2.1. Data Source

The 170Ortho database within PearlDiver (PearlDiver Technologies, Colorado Springs, CO, USA) was used for this retrospective cohort analysis. The dataset encompasses over 170 million patient records from 2010 to 2023 in the United States and complies with the Health Insurance Portability and Accountability Act (HIPAA). The Current Procedural Terminology (CPT) and International Classification of Diseases (ICD) 9th and 10th revision codes were queried to identify patients, diagnoses, procedures, and outcomes. Given the de-identification of patients within the cohorts, this study was deemed exempt from the Institutional Review Board (IRB).

### 2.2. Patient Selection

The RSA cohort was identified using the ICD-9-P-8188, ICD-10-P-0RRJ00Z, and ICD-10-P-0RRK00Z codes. Patients with RA were identified using the following ICD codes: ICD-9-D-714 through ICD-9-D-7142 and ICD-10-D-M05 through ICD-10-D-M059. The cohort of patients undergoing RSA with a prior diagnosis of RA was matched to a control cohort of patients undergoing RSA without a prior diagnosis of RA in a 1:5 ratio. The control group consisted of patients who did not have a prior diagnosis of RA and underwent RSA for any indication including, but not limited to, osteoarthritis, rotator cuff insufficiency, previous failed shoulder arthroplasty, pseudoparalysis, and proximal humerus fractures. These cohorts were then matched to control for age, hypertension, chronic obstructive pulmonary disease, diabetes mellitus, gender, obesity, and tobacco use. 

### 2.3. Outcomes Assessment

Post-operative medical complications within a 90-day period were identified using the corresponding ICD-9 and ICD-10 codes for myocardial infarction, cerebrovascular accident, pulmonary embolism, urinary tract infection, workers’ compensation, acute kidney injury, deep vein thrombosis, post-operative pneumonia, sepsis, blood transfusions, and respiratory failure. Two-year fall rates were assessed with ICD-9-D-E8881 through ICD-9-D-E8889, ICD-10-D-W19XXXA, and ICD-10-D-W19XXXD. The two-year implant-related complications assessed were periprosthetic joint infection, dislocation, mechanical loosening, periprosthetic fracture, osteolysis, and polyethylene wear. Incidence of revision and time from initial shoulder arthroplasty to revision arthroplasty were calculated for both cohorts.

### 2.4. Statistical Analysis

Continuous variables were analyzed using Student’s *t*-tests and are reported as means and standard deviations. Categorical variables were analyzed using chi-squared tests and are reported as frequencies and percentages. Logistic regression analysis was incorporated to assess the difference in medical complications, implant-related complications, fall rates, and readmission rates between both groups. For the results of regression analysis, odds ratios (ORs) at 95% confidence intervals (CIs) were reported. Statistical significance was defined as *p*-value < 0.01. All of the statistical analysis for this study was completed using R, 106 version 4.2.1 software (R Foundation for Statistical Computation, Vienna, Austria).

## 3. Results

An aggregate of 115,042 cases of reverse shoulder arthroplasty were identified, of which 8616 had a diagnosis of RA (7.5%). After matching, 7232 cases of RSA with an RA diagnosis were matched to 36,054 cases of controls. There were no significant differences between the groups for sex, age, and comorbidity burden (Table 1).

### 3.1. Ninety-Day Medical Complications

Patients with RA were significantly more likely to develop all medical complications assessed when compared to matched controls (all *p* < 0.0001) (Table 2). The greatest increase in odds was for urinary tract infection (OR: 9.84, 95% CI: 8.74–11.08, *p* < 0.0001), followed by pulmonary embolism (OR: 9.69, 95% CI: 6.88–13.65, *p* < 0.0001), and the need for blood transfusion (OR: 9.41, 95% CI: 7.74–11.46, *p* < 0.0001).

### 3.2. Two-Year Implant-Related Complications and Falls

Patients with RA who underwent RSA had significantly greater odds of developing all implant-related complications assessed than controls (all *p* < 0.0001) (Table 3). The most substantial increase in odds was for polyethylene wear (OR: 7.84, 95% CI: 4.64–13.23, *p* < 0.0001), followed by osteolysis (OR: 6.17, 95% CI: 3.25–11.7, *p* < 0.0001) and mechanical loosening (OR: 2.53, CI: 2.13–2.99, *p* < 0.0001). Patients in the RA group were significantly more likely to experience falls following surgery compared to controls (OR: 4.22, 95% CI: 3.89–4.78, *p* < 0.0001). 

### 3.3. Length of Stay and Ninety-Day Readmissions

Hospital length of stay was significantly longer in the RA group compared to the control group (3.42 ± 4.73 versus 2.0 ± 1.92 days, respectively; *p* < 0.0001) (Table 4). There were no significant differences in the rates of readmission within 90 days nor in reimbursement between both groups.

## 4. Discussion

Rheumatoid arthritis is an autoimmune condition that is characterized by widespread joint inflammation, synovitis, and a vast array of systemic symptoms. An improved understanding of the pathophysiology and sequalae of RA have prompted noteworthy advancements in its management, specifically through the implementation of disease-modifying anti-rheumatic drugs (DMARDs). In instances of poorly managed disease, erosive arthritis of the peripheral joints, in this case the glenohumeral joint, can be debilitating. Further, patients with RA who are managed with corticosteroids are at risk of developing avascular necrosis of the humeral head [7]. Erosive arthritis and avascular necrosis are both of relevance to shoulder surgeons as they are indications to performing RSA. In addition to the burden on the musculoskeletal system, RA has been associated with a variety of pathologies in other organ systems, most commonly presenting as cardiovascular, respiratory, integumentary, and ocular manifestations [8]. The results of our large-database study demonstrate that RA in RSA patients is associated with a significant risk of post-operative medical and implant-related complications, an increased length of hospital stay, and higher rates of readmission.

There is growing evidence regarding the correlation between complications and RA in total joint arthroplasty. In 2022, Lian et al. [9] demonstrated elevated risk for acute cardiac events, blood transfusions, pulmonary failure, pneumonia, urinary tract infection, prosthetic joint infection, mechanical loosening, and instability following total hip arthroplasty in RA patients versus controls. Further, Jauregui et al. [10] demonstrated an elevated risk of post-operative bleeding requiring transfusion as well as an increased hospital stay for patients with RA who underwent total knee arthroplasty. These findings are similar to those of our study. We found a heightened risk for all medical complications, with urinary tract infection, pulmonary embolism, and blood transfusion having the greatest increase in odds. The existing literature regarding implant-related complications following total joint arthroplasty in RA patients is conflicting [11,12]. Ravi et al. [6] stated higher rates of dislocation following total hip arthroplasty and higher rates of infection following total knee arthroplasty in patients with RA compared to the general population. These findings support our results. On the contrary, Mangold et al. [13] reported a 97% reoperation-free survival at 2 years following RSA in patients with RA. Similarly, Levigne et al. [14] demonstrated low risk of complications and revisions in RA patients undergoing RSA. However, both of these studies are limited by a considerably small sample size, putting the validity of their results into question. Numerous factors play a role in the medical and implant-related complications experienced by RA patients, as evidenced in our study. A chronic inflammatory state has been associated with cytokine-induced damage to blood vessels as well as accelerated plaque formation [15]. This, in combination with the stress of surgery, increases the likelihood of cardiovascular complications such as myocardial infarction and thrombosis. Systemic inflammation and chronic use of glucocorticoids may diminish bone quality, resulting in a greater occurrence of implant-related complications [7,16]. Weakened bone density can compromise secure implant placement and increase the risk of loosening or fracture. RA can cause substantial joint deformity which further contributes to the complexity of optimal implant positioning. The autoimmune nature of this disease delays proper healing and results in a greater propensity for tissue breakdown or dehiscence [17]. Anemia of chronic disease and medication-induced bone marrow suppression increases the likelihood that patients will need blood transfusions post-operatively. Additionally, the use of immunosuppressive drugs in these patients poses a heightened risk of infection [18]. Concerning total shoulder arthroplasty, Boufadel et al. [7] reported that RA was an independent risk factor for 90-day medical complications including myocardial infarction, pneumonia, acute renal failure, and urinary tract infection. Notably, that study did not stratify medical complications based on reverse or anatomic total shoulder arthroplasty. In the same study, RA patients were more likely than controls to encounter periprosthetic joint infection and dislocation following RSA. On the contrary, a systematic review conducted by Cho et al. [19] demonstrated no difference in implant-related complication rates between patients with RA and rotator cuff arthropathy following RSA. Our findings for RSA are consistent with those of Boufadel et al. [7], such that we found an increased risk for all implant-related complications. Our results indicate that polyethylene wear, osteolysis, and mechanical loosening are the implant-related complications with the greatest increase in odds for this subset of patients. The additional medical and implant-related complications observed in patients with RA represent a considerable financial burden to the healthcare system.

Patients with RA should be optimized prior to surgery and strictly monitored post-operatively in efforts to decrease the risk of complication, length of stay, and cost of care. The mean length of stay for RA patients was more than one day longer compared to patients in the control group of our study. Higher complication rates along with the increased need for blood transfusions both contribute to an extended hospital stay. Implementing strategies to reduce the risk of intra- or post-operative blood transfusions should be considered for this subset of patients. Further, pre-operative anemia management involving the assessment of iron levels or supplementation with erythropoietin therapy may be of substantial benefit. Other strategies such as the administration of intra-operative tranexamic acid or the use of blood/cell salvage devices should also be considered to reduce the necessity of blood transfusions. As our results demonstrate that these patients have an elevated risk of infection, further precautions should be taken to address this. This may include a pre-operative discussion with a rheumatologist regarding the adjustment of immunosuppressive medications. Further explanations of the extended hospital stays include the fact that pre-operative joint damage may result in reduced joint mobility and muscle weakness, requiring a longer rehabilitation period. Early mobilization and improved pain management may aid in accelerating the rehabilitation process and reducing the risk of thrombosis. Physicians should have a low threshold of suspicion for the development of acute infection or deep venous thrombosis in these patients, and, thus, strict post-operative monitoring should be implemented. According to the Kaiser Family Foundation [20] the average cost in the United States for one night in the hospital is about $3025. Healthcare expenditures are expected to increase by 5% annually extending into the 2030s [21]. As the prices of healthcare continue to rise, the extended mean hospital stay observed in patients with RA following RSA is noteworthy.

Several studies have established a link between RA and an increased risk of falling [22,23,24]. Disease-related pain, joint deformity, reduced muscle mass, alterations in gait pattern, medications, and decreased functional stability have all been described as contributors to the risk of falling in patients with RA [22]. Our study demonstrated that patients with RA have a four-fold increase in the odds of having a fall following an RSA compared to controls. Modifications in patient care can be made to decrease these risks, such as strict patient monitoring in the early post-operative period and improved living conditions. Further, patient optimization before surgery to increase muscle strength and decrease the utilization of sedating medications may help to counter the risks of falling. 

This is a database retrieval study that is subject to limitations. There is potential for human error in data input and extraction from the database. Additionally, individual databases may not be representative of the entire population. Patient-reported outcomes, which have been shown to be of great value when assessing post-operative outcomes, are lacking, as this is a database study. Due to the nature of the study design, we were not able to accurately identify when a revision procedure was performed and for which patient; therefore, additional statistical analyses such as a Kaplan–Meier survivorship curve could not be reported. Lastly, data were not collected regarding surgical approach, surgeon expertise, implant manufacture, or classification of glenoid deformity. These factors may influence the outcomes collected in this study. A large sample size serves to improve the reliability and generalizability of the results, although this may not always be possible. Additionally, in efforts to reduce any confounding bias, both study groups were matched appropriately to account for a variety of patient factors.

The current data demonstrate an increased risk of medical and implant-related complications following reverse total shoulder arthroplasty in patients with rheumatoid arthritis. The results of this study can provide surgeons and patients with a better idea of post-operative expectations. This may be particularly useful in the aid of constructing treatment plans for this subset of patients. There is a strong financial burden that should be considered when operating on this subset of patients. Optimizing patients with RA prior to surgery and implementing strict post-operative patient monitoring may aid in decreasing costs and complications. 

## Figures and Tables

**Table 1:** A comparison of demographic data and comorbidities between patients with rheumatoid arthritis and a matched-control cohort undergoing reverse shoulder arthroplasty.

Patient Demographics	Rheumatoid Arthritis (RA) RSA	Control (No RA) RSA	*p*-Value
	N = 7232	N = 36,054	
	n (%)	n (%)	
Age (years) *	66.75 ± 6.59	66.79 ± 6.54	0.641
**Sex:**			1.000
Women	5471 (75.65)	27,291 (75.69)	
Men	1761 (24.35)	8763 (24.31)	
**Comorbidity:**			
Hypertension	6821 (94.32)	34,057 (94.46)	0.646
COPD	3751 (51.87)	18,692 (51.84)	0.983
Diabetes Mellitus	3721 (51.45)	18,545 (51.44)	0.992
Obesity	3742 (51.74)	18,628 (51.67)	0.917
Tobacco	3886 (53.73)	19,398 (53.80)	0.924

* given as mean ± standard deviation. RSA, reverse shoulder arthroplasty; RA, rheumatoid arthritis; COPD, chronic obstructive pulmonary disease.

**Table 2:** A comparison of 90-day medical complications between patients with rheumatoid arthritis (RA) and a matched-control cohort undergoing reverse shoulder arthroplasty.

Complication	RA (%)	Control (%)	OR	95% Cl	*p*-Value
Myocardial infarction	1.71	0.30	5.8063	4.48, 7.52	<0.0001
Cerebrovascular accident	1.47	0.19	7.6467	5.65, 10.35	<0.0001
Pulmonary embolism	1.33	0.14	9.6872	6.88, 13.65	<0.0001
Urinary tract infection	11.02	1.24	9.8436	8.74, 11.08	<0.0001
Worker’s compensation	1.34	0.36	3.8157	2.93, 4.97	<0.0001
Acute kidney injury	4.99	0.88	5.9043	5.10, 6.88	<0.0001
Deep vein thrombosis	3.64	0.94	3.9759	3.38, 4.68	<0.0001
Post-op pneumonia	6.18	1.06	6.1684	5.37, 7.09	<0.0001
Sepsis	3.40	0.63	5.5576	4.63, 6.67	<0.0001
Blood transfusion	3.95	0.44	9.4143	7.74, 11.46	<0.0001
Respiratory failure	3.18	0.80	4.0509	3.40, 4.83	<0.0001

**Table 3:** A comparison of two-year falls and implant-related complications between reverse shoulder arthroplasty patients who had rheumatoid arthritis (RA) and a matched-control cohort.

Complication	RA (%)	Control (%)	OR	95% Cl	*p*-Value
Falls after	15.75	4.24	4.2210	3.89, 4.78	<0.0001
Periprosthetic joint infection	3.65	1.96	1.8942	1.64, 2.19	<0.0001
Dislocation	4.94	2.74	1.8411	1.63, 2.08	<0.0001
Mechanical loosening	2.82	1.13	2.5297	2.13, 2.99	<0.0001
Periprosthetic fracture	1.47	0.80	1.8345	1.46, 2.29	<0.0001
Osteolysis	0.29	0.05	6.1734	3.25, 11.7	<0.0001
Polyethylene wear	0.50	0.06	7.8372	4.64, 13.23	<0.0001

**Table 4:** A comparison of healthcare utilization and reimbursement amounts between patients with rheumatoid arthritis (RA) and a matched-control cohort undergoing reverse shoulder arthroplasty.

Utilization	RA	Control	OR	95% CI	*p*-Value
Readmission	6.60%	5.90%	1.1258	1.06, 1.25	0.0237
Length of Stay (days) *	3.42 ± 4.73	2.00 ± 1.92			<0.001
Reimbursement ($) *	5636.65 ± 9601.71	5829.35 ± 9425.80			0.118

* given as mean ± standard deviation. OR, odds ratio; CI, confidence interval.

## Data Availability

The original contributions presented in this study are included in the article. Further inquiries can be directed to the corresponding author.
